# A novel chemoreactive calcilytic for the potential treatment of autosomal dominant hypocalcemia

**DOI:** 10.1016/j.apsb.2025.07.044

**Published:** 2025-08-07

**Authors:** Jesse Dangerfield, Aaron DeBono, Andrew N. Keller, Tracy M. Josephs, David M. Shackleford, Karen J. Gregory, Katie Leach, Ben Capuano

**Affiliations:** a Medicinal Chemistry, Monash Institute of Pharmaceutical Sciences, Monash University, VIC 3052, Australia; b Drug Discovery Biology, Monash Institute of Pharmaceutical Sciences, Monash University, VIC 3052, Australia; c Australian Research Council Centre for Cryo-electron Microscopy of Membrane Proteins, Monash University, VIC 3052, Australia; d Centre for Drug Candidate Optimisation, Monash Institute of Pharmaceutical Sciences, Monash University, VIC 3052, Australia

**Keywords:** Calcium-sensing receptor, Calcilytic, Chemoreactive, Irreversible, Autosomal dominant hypocalcemia

## Abstract

Autosomal dominant hypocalcemia (ADH) type 1 and 2 are disorders of calcium homeostasis caused by gain of function variants. The calcium-sensing receptor (CaSR) is a class C GPCR that responds to elevated extracellular calcium (Ca^2+^_o_) by inhibiting parathyroid hormone (PTH) secretion and promoting renal excretion of Ca^2+^ and other salts to restore physiologically normal Ca^2+^_o_ concentrations. CaSR negative allosteric modulators (NAMs) transiently raise PTH levels in individuals with ADH1, restoring Ca^2+^_o_ concentration to a physiological normal range. Herein we disclose the discovery of a chemoreactive NAM (ATF936-NCS, **4**) for the CaSR that (i) is wash-resistant indicative of irreversible receptor binding and (ii) stimulates prolonged PTH release *in vivo*. This ‘first-in-class’ chemical probe will provide invaluable insight towards the development of longer acting NAMs for the treatment of ADH.

## Graphical abstract

The discovery of a first-in-class chemoreactive CaSR NAM derived from ATF936 that demonstrates a unique receptor binding mechanism and a sustained PTH release profile of therapeutic potential.

**Figure:**
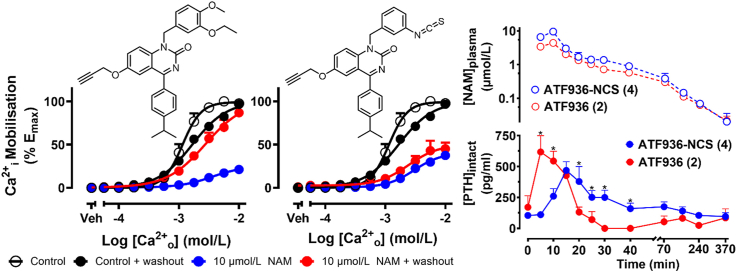

## 1 Introduction

**Figure 1:**
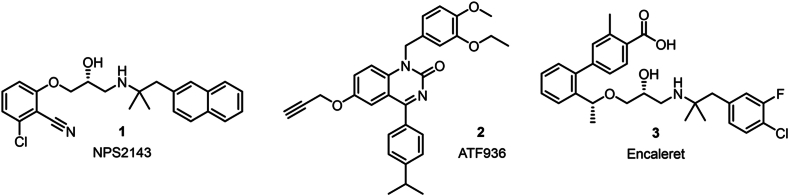
Chemical structures of two key chemical classes of CaSR NAMs: amino-alcohols (NPS2143, **1** and encaleret, **3**) and 2-quinazolinones (ATF936, **2**).

The calcium-sensing receptor (CaSR) is a class C GPCR that responds to elevated extracellular calcium (Ca^2+^_o_) by inhibiting parathyroid hormone (PTH) secretion and promoting renal excretion of Ca^2+^ and other salts to restore physiologically normal Ca^2+^_o_ concentrations. Autosomal dominant hypocalcemia types 1 (ADH1) and 2 (ADH2) are rare disorders of extracellular calcium (Ca^2+^_o_) homeostasis^1^^,^^2^, caused by gain-of-function variants in the CaSR^3^ and G protein subunit alpha 11 (G_*α*11_)^2^^,^^4^, respectively. ADH causes hypocalcemia with reduced PTH secretion and normal or increased urinary Ca^2+^_o_ excretion (hypercalciuria). ADH symptoms, including paraesthesia, painful muscle spasms with sharp flexion of the wrists and ankles, and recurrent childhood seizures^5^^,^^6^^,^^7^,occur in approximately 50% of people living with ADH^7^. CaSR NAMs are promising ADH treatments and comprise two main chemical classes of orally bioavailable compounds; the amino-alcohols such as NPS2143 (**1**) and quinazolinones like ATF936 (**2**) (Fig. 1)^8^. CaSR negative allosteric modulators (NAMs) transiently raise PTH levels in people with ADH1^9^ restoring Ca^2+^_o_ concentration to a physiological normal range. While CaSR NAMs have been described to date, none have progressed beyond clinical trials as these NAMs were specifically designed to treat osteoporosis by transiently increasing PTH levels. Recently computational screening efforts have been undertaken in the interest of identifying novel chemotypes for the CaSR with the need of unlocking new pharmacological profiles^10^. Currently encaleret (**3**) from the amino alcohol class of NAMs is an exciting prospect in the treatment of ADH and has reached phase 3 clinical trials^11^. Literature suggests, these amino alcohol NAMs are generally unsuitable for all individuals with ADH due to non-response commonly associated with pharmacogenetic effects of ADH variants, which reduce NAM activity due to compromised binding sites^12^.

Cellular studies show CaSR NAMs rectify *in vitro* alterations in CaSR signaling caused by gain-of-function CaSR and G*α*_11_ variants^13^^,^^14^. *In vivo* studies demonstrate a transient increase in blood Ca^2+^_o_ concentrations and prevention of nephrocalcinosis in ADH1 knock-in mice expressing mutant CaSR to mimic ADH1^15^^,^^16^. The same transient increase in PTH in humans with ADH1 is seen but with no alteration in blood Ca^2+^_o_ concentrations^9^. The pharmacokinetic profile of currently available NAMs, however, is not appropriate for the treatment of ADH because these drugs were optimized to have a short *in vivo* half-life to transiently increase PTH secretion to induce bone anabolic effects conducive to bone formation in osteoporosis^17^. Optimal ADH treatment requires a long-acting NAM to promote sustained elevations in PTH and blood Ca^2+^_o_ concentrations.

In 2010, Novartis disclosed the CaSR NAM ATF936 during a drug discovery program for novel osteoporosis therapeutics. ATF936 is a potent NAM with good oral bioavailability and PTH release when dosed to rats and dogs^18^. Previous studies demonstrated both ATF936 and the related quinazolinone NAM ATX914, dosed in healthy male patients, were safe, but the compounds failed to progress due to a lack of efficacy and inability to increase bone mineral density. While these NAMs were unable to be used for osteoporosis, recent work explored repurposing for treating ADH^19^. Calcilytics used in both mice models and humans induced increased PTH levels, with a rapid on- and off-effect of PTH release, highlighting potential as future long term ADH treatments.

As a lifelong disease, ADH is linked to a number of complications with more than 35% of people with ADH develop ectopic calcifications of the basal ganglia and kidneys^5^^,^^20^. Individuals with severe forms of ADH1 develop a Bartter-like syndrome characterized by hypocalcemic alkalosis, renal salt wasting and hyperreninemic hyperaldosteronism^21^^,^^22^. ADH has a high clinical unmet need because conventional therapies such as vitamin D analogues and calcium supplements cause excessive activation of renal CaSRs, aggravating hypercalciuria and predisposing people with ADH to nephrocalcinosis and renal failure^5^^,^^20^. Recombinant PTH (rPTH) is occasionally used to treat ADH but does not prevent development of renal complications^23^.

Many of the therapeutics developed for the aforementioned disease focus on the principle of a non-covalent, reversible interaction with the receptor of interest in order to achieve a transient PTH release profile. Historically, there has been hesitance to use chemoreactive groups in small molecule inhibitors largely around concerns about toxicity. Recently, covalent ligands that inherently form a bond with their target protein have emerged as an attractive therapeutic strategy. Targeted covalent inhibitors have been approved for the treatment of cancers such as Ibrutinib and Osimertinib, with current advancements seeking to broaden both the reactive functional groups and targeted residues used and to expand into disease states beyond cancers^24^^,^^25^. Here, we report the discovery of the first covalent negative allosteric modulator targeting CaSR based on the existing calcilytic agent ATF936.

## 2 Results and discussion

### 2.1 Docking of ATF936 and ATF936 derivatives into a cryo-EM based model of the human CaSR

**Figure 2:**
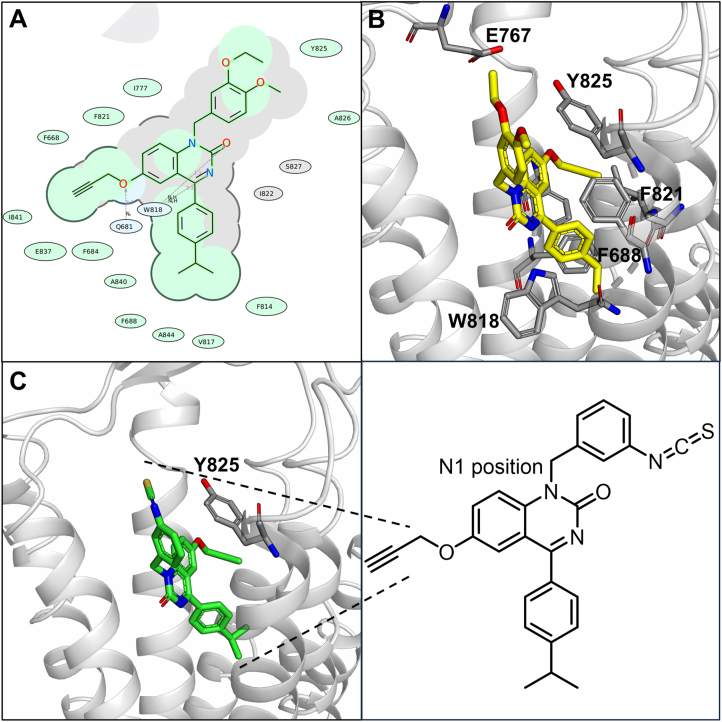
Predicted comparative docking poses of the parent compound (**2**) and the chemoreactive reactive variant (**4**) (A) 2D binding pocket of ATF936 (**2**) in the CaSR 7TM where green shading represents hydrophobic regions; blue represents hydrogen bond acceptors and grey represents accessible surface area; (B) Proposed binding mode of ATF936 in the CaSR 7TM in an inactive model derived from the Cryo-EM structure PDBID:7M3E^28^ (note that the isopropyl group is eclipsed); (C) Proposed binding mode of ATF936-NCS (**4**) with residue Y825 highlighted (with accompanying chemical structure).

All small molecule CaSR allosteric modulators to date bind to an allosteric binding pocket located towards the top of the transmembrane domains^26^^,^^27^. ATF936 was docked into a model derived from the inactive Cryo-EM CaSR structure 7M3E with NPS2143 bound, in order to predict the novel NAM CaSR binding mode and identify potential residues available for covalent interaction (Fig. 2)^28^. ATF936 is predicted to form several key interactions with the receptor: *e.g.* two hydrogen bonds, one between the nitrogen of the 2-quinazolinone with W818 and the second between the aryl ether oxygen with Q681. The propargyl group is key for affinity and sits within a small hydrophobic pocket. The cumene is located towards the bottom of the binding pocket surrounded by hydrophobic residues F688, F814, V817. The substituted benzyl substituent at the N1 position of the 2-quinazolinone extends towards extracellular space in two different equal energy conformations, supporting previous SAR work showing this position is tolerant to a range of functional groups.

**Figure 3:**
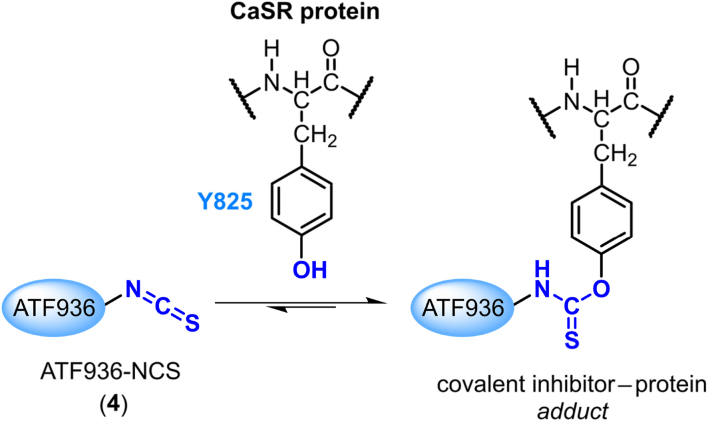
Predicted covalent reaction between an electrophilic isothiocyanate warhead and a nucleophilic tyrosine residue to form a reversible thiocarbamate-linked adduct.

The N1 position tolerates a range of different substituents and as such presents an ideal location for a chemoreactive moiety^29^. Upon docking of **2**, the closest residue able to participate in covalent interactions with a substitution on the N1 benzyl group was the phenolic sidechain of the tyrosine residue, Y825. Sulfonyl fluoride, isothiocyanates and haloacetamides are chemoreactive groups known to bind covalently to tyrosine residues, however, of particular interest was the isothiocyanate group which can act as a reversible covalent group, potentially circumventing future toxicity concerns^30^^,^^31^. ATF936 analogues containing the aforementioned chemoreactive groups were docked, the 3′-position isothiocyanate was particularly promising with a distance of 4.1 Å between the carbon of the isothiocyanate functional group and the phenolic oxygen of Y825. The 2′- and 4′-position isothiocyanate derivatives were also docked but the isothiocyanate residue was not predicted to be within range of the reactive residue to form a covalent interaction (see binding poses in Supporting Information, Supporting Information Figs. S1 and S2). Tyrosine and isothiocyanate may react covalently to form the *O*-thiocarbamate, thus allowing for prolonged receptor inhibition and reduced off-target effects compared to traditional reversible inhibitors (Fig. 3).

### 2.2 Chemical synthesis of ATF936-NCS (**4**)

**Scheme 1:**
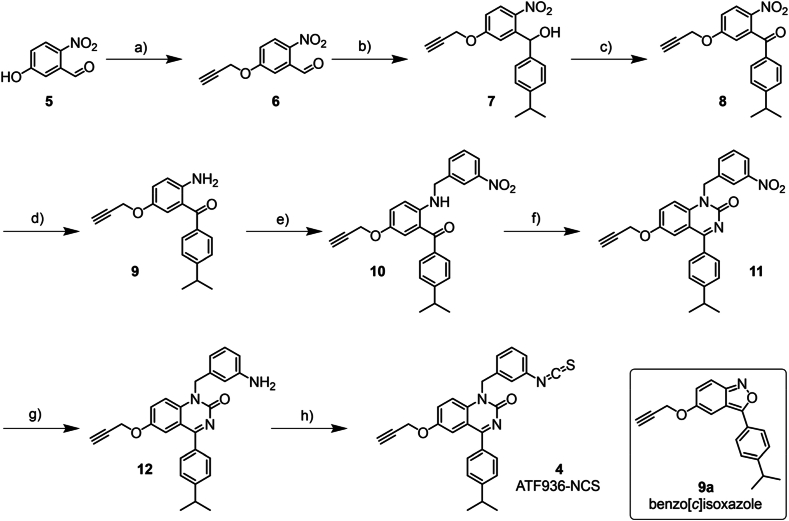
Chemical synthesis of intermediate compounds **5**–**12** and ATF936-NCS (4). Reagents and conditions: a) propargyl bromide, K_2_CO_3_, DMF, 15 h, 84%; b) 4-isopropylphenyl magnesium bromide, anhydrous THF, N_2,_ 0 °C, 12 h, 59%; c) 2,2,6,6-tetramethyl-1-piperidinyloxyl, (diacetoxyiodo)benzene, DCM, rt, 20 h, 98%; d) SnCl_2_, abs. EtOH, 80 °C, 48 h, 74%; e) 3-nitrobenzyl bromide, DIPEA, 1,4-dioxane, 80 °C, 24 h; f) NaOCN, glacial AcOH, rt, 12 h, 36% over two steps; g) SnCl_2_, EtOH, 80 °C, 48 h, h) TCDI, DMAP, THF, N_2_, 2 h, 34% over two steps.

Due to the high inherent reactivity of chemoreactive groups, late-stage introduction of the relevant functional group is key to limiting degradation or side reactions. With this in mind, the synthetic scheme was adapted from work by Wilder et al.^29^ to enable alkylation with 3-nitrobenzyl bromide to furnish a late-stage nitro substituent, which following reduction to the primary aromatic amine, this allowed facile conversion to the desired isothiocyanate group. Synthesis began with the alkylation of 5-hydroxy-2-nitrobenzaldehyde **5** with propargyl bromide under basic conditions. The corresponding *O*-alkylated product **6** was then reacted with the Grignard reagent 4-isopropyl phenylmagnesium bromide to convert the aldehyde functional group to the corresponding secondary alcohol **7**. The desired ketone **8** was previously furnished from the respective secondary alcohol using oxidizing agents like Jones reagent^29^, but to avoid use and disposal of this known carcinogen, the reaction proceeded with the reagents 2,2,6,6-tetramethyl-1-piperidinyloxyl (TEMPO) and (diacetoxyiodo)benzene (BAIB) without compromising yield. To avoid reduction of the alkyne moiety, catalytic hydrogenation was avoided. Initial attempts at the nitro reduction using a Bechamp reduction (Fe, HCl) returned only starting material, whilst two methods using zinc with either formic acid or ammonium chloride yielded solely a benzo[*c*]isoxazole product (**9a**), formed by intramolecular cyclisation. Due to its inherent similarity to the target quinazolin-2-one core, **9a** was tested but showed no NAM activity (data not shown). The nitro group was successfully reduced using tin(II) chloride to yield the aniline **9**; as the aniline is deactivated by the *ortho* ketone, the reaction required forcing conditions at prolonged reaction times. Cyclisation of the substituted aniline **9** to form the 2-quinazolinone **11** was performed using sodium cyanate in glacial acetic acid. The newly installed nitro group was reduced to the corresponding aniline **12** using the previously described nitro reduction method, before being reacted on without further purification (due to stability issues) with TCDI and DMAP to give the desired chemoreactive isothiocyanate derivative (ATF936-NCS, **4**) in good yield (Scheme 1, see characterization data in Supporting Information, Supporting Information Figs. S3.1–S3.4).

### 2.3 Pharmacological characterization of ATF936-NCS (**4**) shows retained NAM activity

#### 2.3.1 Intracellular (Ca^2+^_i_) mobilization assay

**Figure 4:**
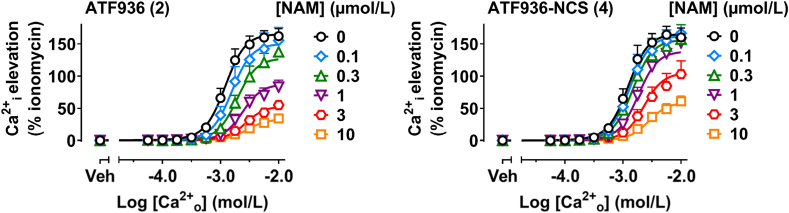
Pharmacological evaluation of ATF936 (**2**) and ATF936-NCS (**4**) in FlpIn HEK TRex cells stably expressing the CaSR using Ca^2+^_i_ mobilization assays. Data reported are mean ± SEM from multiple independent experiments performed in duplicate, and have been normalized to ionomycin. Curves through the data are the best fit of the complete operational model of cooperative agonism and allosterism (Equation 1), used to quantify NAM p*K*_B_ and *αβ*.

The pharmacology of **4** was assessed in Ca^2+^_i_ mobilization assays in Flp-In HEK293-TREx cells (Fig. 4). ATF936 (**2**) and ATF936-NCS (**4**) were pre-incubated with the cells prior to the addition of Ca^2+^_o_. By running this assay at a broad range of compound and calcium concentrations (Fig. 4), an operational model of cooperative agonism and allosterism^32^ can be applied to quantify; (i) the negative logarithm of ligand binding affinity for the allosteric site (p*K*_B_) and (ii) the composite cooperativity parameter (*αβ*) as a global measure of the allosteric ligand's effect on Ca^2+^_o_ affinity (*α*) and efficacy (*β*) (Table 1). Compound **4** demonstrated sub μmol/L affinity for the CaSR (*K*_B_ = 407 nmol/L), with an approximate 5-fold reduction in affinity compared to the reference compound **2** (*K*_B_ = 87 nmol/L). The lower affinity of **4** relative to **2** can be attributed to steric effects as formation of the *O*-thiocarbamate may force **4** to adopt a less favorable conformation. Noteworthy, compound **4** retained NAM activity, and serves as a proof of concept for correcting ADH with CaSR chemoreactive NAMs.

**Table 1:** Chemical structures, p*K*_B_, Log*αβ* and *K*_B_ values derived from Ca^2+^_i_ mobilization assays.

Compound	Chemical structure	p*K*_B_ ± SEM (*n*)	*K*_B_ (nmol/L)	Log*αβ* ± SEM (*αβ*)
ATF936 (**2**)		7.06 ± 0.13 (**4**)	87	−1.87 ± 0.22 (0.0135)
ATF936-NCS (**4**)		6.39 ± 0.16 (**4**)	407	−2.38 ± 1.31 (0.00417)

The curve fits for the operational model used had difficulty fitting the higher drug concentrations of ATF936-NCS and to a lesser extent ATF936. Using the standard 4-parameter model it became clear that the Hill slope was decreasing as compound concentration increased (Table 2). The steep Hillslope of Ca^2+^_o_ CaSR activation is thought to be due to a combination of multiple Ca^2+^_o_ binding sites having cooperativity and the naturally dimeric nature of the CaSR^32^. The observed change in Hill slope may be caused by differences in Ca^2+^_o_ occupancy within and across the dimer or allosteric modulation of cooperative Ca^2+^_o_ binding. Although the initial model is an imperfect fit, the values derived can be used as estimates as the compounds are clearly acting as NAMs.

**Table 2:** LogEC_50_, *E*_max_ and Hill slope values derived from Ca^2+^_i_ mobilization assays for each concentration of ATF936 (**2**) and ATF936-NCS (**4**) fit to a 4-parameter nonlinear regression of log agonist *vs* response (Eq. 3).

[NAM] (μmol/L)	ATF936 (2)			ATF936-NCS (4)			*n*
	LogEC_50_	*E*_max_	Hill slope	LogEC_50_	*E*_max_	Hill slope	
0	−2.94 ± 0.06	161.3 ± 10.4	2.91 ± 0.06	−2.93 ± 0.07	163.6 ± 13.7	3.39 ± 0.43	4
0.1	−2.74 ± 0.13	159.4 ± 14.2	2.53 ± 0.29	−2.85 ± 0.08	163.5 ± 13.8	2.73 ± 0.17	4
0.3	−2.66 ± 0.06∗	138.6 ± 13.1	2.35 ± 0.08	−2.81 ± 0.10	155.8 ± 20.7	2.63 ± 0.16	4
1	−2.62 ± 0.08∗	84.5 ± 9.1∗	2.41 ± 0.24	−2.71 ± 0.05	151.4 ± 16.1	2.37 ± 0.07∗	4
3	−2.45 ± 0.08∗	62.4 ± 4.6∗	1.89 ± 0.13∗	−2.58 ± 0.05∗	108.3 ± 20.3	2.00 ± 0.17∗	4
10	−2.43 ± 0.04∗	41.2 ± 5.2∗	1.62 ± 0.06∗	−2.50 ± 0.04∗	66.9 ± 7.7∗	1.92 ± 0.18∗	4

Data reported are mean ± SEM from multiple independent experiments performed in duplicate and have been normalized to ionomycin. Curves through the data are the best fit of a 4-parameter nonlinear regression of log agonist *vs* response (Eq. 3). *E*_max_ is expressed as % ionomycin response. ∗*P* < 0.05 where statistical significance compared to [NAM] = 0 was determined by one-way ANOVA with Dunnett's post-test.

#### 2.3.2 Washout assay to determine extended NAM activity

**Figure 5:**
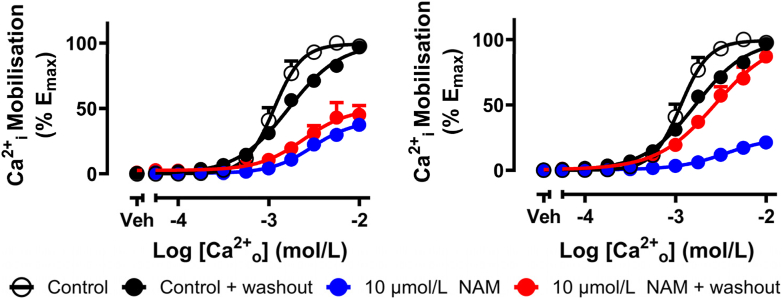
CaSR-mediated Ca^2+^_i_ elevation in HEK293-CaSR cells following a 30 min preincubation with vehicle/NAM or NAM with 3 × 1 h washes. Curves through the data are the best fit of a 4-parameter nonlinear regression of log agonist *vs* response (equation 3). Data was normalized to the maximal response of ionomycin and then normalized to the maximal response of Ca^2+^_i_. *E*_max_ is expressed as the % Ca^2+^ response. *n* = 3 for 10 μmol/L + washout, *n* = 4 for 10 μmol/L response (shown earlier in Fig. 4).

A washout assay was used to determine whether the chemoreactive compound irreversibly bound the CaSR. The chemoreactive derivative ATF936-NCS (**4**) and reference compound ATF936 (**2**) were independently preincubated with cells for 2 h to enable irreversible linkage before a series of washes to remove any non-irreversibly linked modulator from the receptor. We then assessed Ca^2+^_i_ elevation in response to Ca^2+^_o_ (Fig. 5). ATF936 pre-treatment followed by washout had no effect on Ca^2+^_i_ elevation, suggesting ATF936 had been successfully washed out. At 10 μmol/L, the chemoreactive derivative **4** inhibited the Ca^2+^_o_ response after washout, suggesting **4** was irreversibly linked with the receptor. This observation doesn't preclude that the inability to wash out **4** from the receptor may be due to a slow off-rate of the ligand instead of irreversible binding, however, either result gives the desired profile.

#### 2.3.3 *In vivo* administration of ATF936-NCS stimulates sustained PTH release

**Figure 6:**
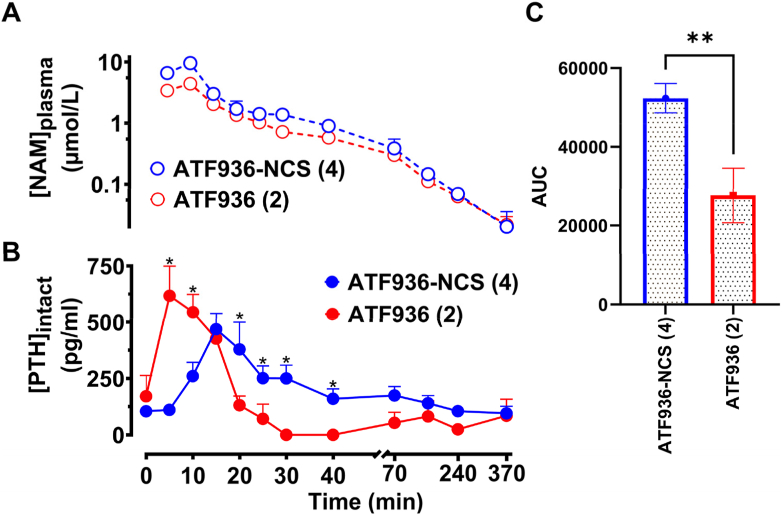
*In vivo* PTH release profile of the novel chemoreactive derivative (**4**) compared to the reference compound, ATF936 (**2**). (A) ATF936-NCS (**4**) or ATF936 (**2**) plasma concentrations are equivalent following a 10-min intravenous infusion of 0.61 mg/kg; *n* = 3 rats per compound. (B) **4** stimulates distinct elevations in PTH concentrations compared to **2** (*n* = 3, ∗*P* < 0.05 where statistical significance compared to **2** was determined by two-way repeated measures ANOVA with multiple comparisons). (C) Area under the curve analysis of **B** demonstrates a significant difference in PTH release over time for **4** compared to **2** (∗∗*P* < 0.01 where statistical significance compared to **2** was determined by an unpaired two-tailed *t*-test).

To ascertain whether an irreversible CaSR NAM would translate into extended release of PTH, Sprague-Dawley rats were independently administered ATF936 (**2**) or the chemoreactive derivative ATF936-NCS (**4**) as a 10-min intravenous infusion (0.61 mg/kg; *n* = 3 rats per compound). Samples of blood were taken at the middle and end of the infusion (5 and 10 min) and then intermittently to 370 min (15, 20, 25, 30, 40, 70, 160, 240 and 370 min). **2** and **4** demonstrated similar plasma concentration profiles, each reaching a maximum plasma concentration at 10 min (end of infusion) before exhibiting very similar rates of decline (Fig. 6A).

A marked difference, however, was observed in PTH levels, with release both slower to peak and more sustained in rats administered the isothiocyanate, ATF936-NCS (**4**), as evident in the plasma PTH concentration-time profile (Fig. 6B). ATF936 reached maximal PTH levels after 5 min during the infusion phase before rapidly dropping towards zero within 30 min, whereas ATF936-NCS (**4**) achieved maximal PTH levels after 15 min before slowly decreasing to basal levels after 6 h. This noticeable difference is highlighted by the almost 2-fold increase in PTH release for ATF936-NCS (**4**) over that of ATF936 (**2**) as illustrated by the relative AUC determination (Fig. 6C). The distinct difference in PTH concentration profile of ATF936-NCS (**4**) compared to ATF936 (**2**) is likely due to irreversible binding. The slower peak of PTH release is possible due to the differences in distinct binding kinetics of the irreversible versus reversible NAM, resulting in a slower onset of action. These data provide support for the concept that irreversible CaSR NAMs with sustained PTH release profiles may overcome the limitations linked to short duration of action and potentially offer a strategy toward viable treatments to correct the associated symptoms of ADH.

#### 2.3.4 ATF936-NCS demonstrates superior NAM activity compared to NPS2143 at an ADH1 causing mutation

**Figure 7:**
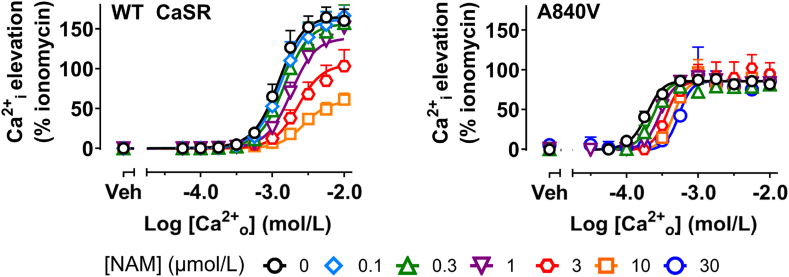
Ca^2+^_o_-mediated Ca^2+^_i_ elevation in HEK293 cells stably expressing the wild type (WT) CaSR (shown earlier in Fig. 4) or an ADH1 A840V CaSR mutant in the absence or presence of varying concentrations of ATF936-NCS (**4**). Curves through the data are the best fit of an operational model of cooperative agonism and allosterism (equation 1 or equation 2), used to quantify NAM p*K*_B_ and *αβ*. *n* = 4 for ATF936-NCS (**4**) WT and A840V.

In an ADH1 human clinical trial, an individual harboring an A840V CaSR mutation showed reduced elevations in PTH in response to NPSP795^9^. Here with the CaSR A840V mutation expressed in HEK293 cells, the affinity and cooperativity of the amino-alcohol NAM are reduced in Ca^2+^_i_ mobilization assays (Table 3, Fig. 7)^19^^,^^12^. When tested in the same mutant, the chemoreactive compound **4** demonstrated superior cooperativity to amino-alcohol NAMs such as NPS2143 (**1**) and consequently overcomes mutation-induced gains in receptor function more readily (Fig. 7). Due to the large number of variants that can cause ADH, it may be necessary to have multiple NAMs with different chemotypes as treatment options for individuals whose variants may make them unresponsive to a single chemical class. This demonstrates how pharmacogenetic effects detected *in vitro* may translate to differences in patient responsiveness to drugs in the clinic.

**Table 3:** CaSR NAM p*K*_B_, log*αβ* and *K*_B_ values derived from Ca^2+^_i_ mobilization assays at both the wildtype and A840V variant.

Compound	WT			A840V^a^		
	p*K*_B_	*K*_B_ (nmol/L)	Log*αβ* (*αβ*)	p*K*_B_ ± SEM (*n*)	*K*_B_ (nmol/L)	Log*αβ* ± SEM (*αβ*)
NPS2143 (**1**)^b^	7.33	47	−1.73 (0.019)	6.76	174	−0.95 (0.112)
ATF936-NCS (**4**)	Refer to Table 1			6.65 ± 0.23 (4)	224	−2.40 ± 0.77 (0.004)

a A840V data were best fitted with the comparative version of the operational model of cooperative agonism and allosterism (Eq. 2), whereas WT data (see Table 1) were fitted to the complete operational model of cooperative agonism and allosterism (Eq. 1). Analyses were performed on the collated data to derive the estimates of each parameter.

b Data for NPS2143 were previously reported^26^^,^^32^, the best fit of the data using Eq. 2 are presented here to enable comparison.

## 3 Conclusions

This study reports the first-in-class design, synthesis, and pharmacological characterization of an isothiocyanate-containing and likely irreversible CaSR NAM (ATF936-NCS, **4**). Computational modelling guided design based on a predicted covalent interaction with Y825 through the phenolic O atom. Initial *in vitro* experiments determined the predicted covalent compound retained a NAM pharmacological profile after wash out and *in vivo* tests illustrated ATF936-NCS produced prolonged elevation of PTH levels. Coupled with evidence that this compound class is able to retain a NAM effect in variants that amino alcohol NAMs, like NPS2143 (**1**) fail to do so, suggests that quinazolinone NAMs and specifically chemoreactive species are an exciting prospect in the potential treatment of ADH.

## 4 Experimental

### 4.1 Reagents

Thin layer chromatography (TLC) was performed using TLC silica gel 60 F_254_ aluminium sheets (0.25 mm, Merck). Flash column chromatography was carried out with silica gel 60, 0.6–0.20 mm (70–230 mesh, Merck). ^1^H (400.13 MHz) and ^13^C NMR (100.62 MHz) spectra obtained on a Bruker Avance III Nanobay spectrometer with a BACS 60 sample changer using solvents from Cambridge Isotope Laboratories. Chemical shifts (*δ*, ppm) are reported relative to the solvent peak (CDCl_3_: 7.26 [^1^H] or 77.16 [^13^C]. Coupling constants (*J*) are given in hertz (Hz), and the following abbreviations were assigned for signal multiplicities: brs = broad singlet, s = singlet, d = doublet, t = triplet, and m = multiplet. The purity of the compounds (>95%) was established analytical HPLC. Analytical HPLC was acquired on an Agilent 1260 Infinity analytical HPLC coupled with a G1322 A degasser, G1312 B binary pump, G1367 E high-performance autosampler, and G4212 B diode array detector. Conditions were as follows: Zorbax Eclipse Plus C18 rapid resolution column (4.6 mm × 100 mm) with UV detection at 254 and 214 nm, 30 °C; the sample was eluted using a gradient system, where solvent A was 0.1% aq. TFA and solvent B was 0.1% TFA in CH_3_CN (5%–100% B [9 min],100% B [1 min]; 0.5 mL/min). Low-resolution mass spectra (MS) were run on an Agilent 6100 series single quad LC–MS coupled with an Agilent 1200 series HPLC, G1311 A quaternary pump, G1329 A thermostated autosampler, and G1314 B variable wavelength detector (214 and 254 nm). LC conditions were as follows: Phenomenex Luna C8(2) column (100 Å, 5 μm, 50 mm × 4.6 mm), 30 °C; the sample (5 μL) was eluted using a binary gradient (solvent A: 0.1% aq. HCO_2_H; solvent B: 0.1% HCO_2_H in CH_3_CN; 5%–100% B [10 min], 100% B [10 min]; 0.5 mL/min). MS conditions were as follows: quadrupole ion source with multimode ESI; drying gas temperature, 300 °C; vaporizer temperature, 200 °C; capillary voltage, 2000 V (positive mode) or 4000 V (negative mode); scan range, 100–1000 *m*/*z*; and step size, 0.1 s over 10 min. High-resolution MS was performed on an Agilent 6224 TOF LC–MS coupled to an Agilent 1290 Infinity LC. All data were acquired and reference mass corrected *via* a dual-spray electrospray ionization (ESI) source. Each scan or data point on the total ion chromatogram (TIC) is an average of 13,700 transients, producing a spectrum every second. Mass spectra were created by averaging the scans across each peak and subtracting the background from the first 10 s of the TIC. Acquisition was performed using the Agilent Mass Hunter Data Acquisition software (ver. B.05.00, build 5.0.5042.2), and analysis was performed using Mass Hunter Qualitative Analysis (ver. B.05.00 build 5.0.519.13). Acquisition parameters were as follows: mode, ESI; drying gas flow, 11 L/min; nebulizer pressure, 45 psi; drying gas temperature, 325 °C; voltages: capillary, 4000 V; fragmentor, 160 V; skimmer, 65 V; octapole RF, 750 V; scan range, 100–1500 *m*/*z*; and positive ion mode internal reference ions, *m*/*z* 121.050873 and 922.009798. LC conditions were as follows: Agilent Zorbax SBC 18 rapid resolution HT (2.1 mm × 50 mm, 1.8 μm column), 30 °C; the sample (5 μL) was eluted using a binary gradient (solvent A: 0.1% aq. HCO_2_H; solvent B: 0.1% HCO_2_H in CH_3_CN; 5%–100% B [3.5 min], 0.5 mL/min). All glassware was dried in an oven at 110 °C and cooled under nitrogen gas prior to use.

### 4.2 Synthesis

#### 4.2.1 2.Nitro-5-(prop-2-yn-1-yloxy)benzaldehyde (**6**)^29^

A mixture of 5-hydroxy-2-nitrobenzaldehyde (600 mg, 3.59 mmol), K_2_CO_3_ (992 mg, 7.18 mmol) and propargyl bromide (441 μL, 3.96 mmol) in DMF (5 mL) was stirred at rt for 15 h. The mixture was concentrated *in vacuo*, the dark brown oil was taken up in DCM (10 mL) and washed with water (3 mL). The organic layer was dried with anhydrous Na_2_SO_4_, then concentrated *in vacuo* to give a dark brown amorphous solid which was carried on without further purification (622 mg, 84%). LC–MS (*m*/*z*): 228 [M+Na]^+^; HPLC: *t*_R_ 5.137 min, >95% purity (214 and 254 nm). ^1^H NMR (400 MHz, CDCl_3_) *δ* 10.49 (s, 1H), 8.18 (d, *J* = 8 Hz, 1H), 7.26 (d, *J* = 2.8 Hz, 1H), 7.24 (d, *J* = 2.8 Hz, 1H), 4.84 (d, *J* = 2.4 Hz, 2H), 2.60 (t, *J* = 2.4 Hz, 1H). ^13^C NMR (101 MHz CDCl_3_) *δ* 188.3 (C), 161.9 (C), 143.0 (C), 134.3 (C), 127.3 (CH), 119.5 (CH), 114.6 (CH), 77.4 (C), 76.7 (CH), 56.8 (CH_2_).

#### 4.2.2 (4-Isopropylphenyl) (2-nitro-5-(prop-2-yn-1-yloxy)phenyl)methanol (**7**)^29^

Compound **6** (100 mg, 0.48 mmol) was dissolved in dry THF under a N_2_ atmosphere. 4-Isopropylphenyl magnesium bromide (0.5 mol/L in THF) (1.46 mL, 0.731 mmol) was added dropwise at 0 °C, stirred for 30 min before warmed to rt and stirred for a further 12 h. The reaction was quenched with saturated aqueous NH_4_Cl (2 mL) and extracted with EtOAc (5 mL). The organic layer was dried with anhydrous Na_2_SO_4_ and concentrated *in vacuo*. The resulting residue was purified by flash chromatography using Petroleum spirits and EtOAc (8:1). Concentration of column fractions afforded the product as a dark brown oil (93.2 mg, 59%). LC–MS (*m*/*z*): 348.9 [M+Na]^+^; HPLC: *t*_R_ 6.970 min, >95% purity (214 and 254 nm); ^1^H NMR (400 MHz, CDCl_3_) *δ* 8.06 (d, *J* = 9.2 Hz, 1H), 7.43 (d, *J* = 2.8 Hz, 1H), 7.25 (d *J* = 8.0 Hz, 2H), 7.17 (d, *J* = 8.4 Hz, 2H), 6.97 (dd, *J* = 9.2 Hz, 2.8 Hz, 1H), 6.50 (s, 1H), 4.78 (d, *J* = 2.4 Hz, 2H), 2.88 (sept, *J* = 6.8 Hz, 1H), 2.56 (t, *J* = 4.8 Hz, 2.4 Hz, 1H), 1.22 (d, *J* = 6.8 Hz, 6H). ^13^C NMR (101 MHz CDCl_3_) *δ* 161.5 (C) 148.9 (C), 142.3 (C), 141.6 (C) 139.0 (C), 127.9 (CH), 127.3 (CH), 126.8 (CH), 115.2 (CH), 114.0 (CH), 76.8 (C), 71.7 (CH), 56.4 (CH_2_), 33.9 (CH), 24.0 (CH_3_).

#### 4.2.3 (4-Isopropylphenyl) (2-nitro-5-(prop-2-yn-1-yloxy)phenyl)methanone (**8**)^29^

A reaction mixture of **7** (25 mg, 0.0773 mmol), 2,2,6,6-tetramethyl-1-piperidinyloxyl (1.2 mg, 7.73 μmol) and (diacetoxyiodo)benzene (27.4 mg, 0.085 mmol) dissolved in DCM (2 mL) was stirred for 20 h at rt. The reaction mixture was diluted with DCM (5 mL), washed with satd. aqueous Na_2_SO_3_ (5 mL) and back-extracted with DCM (2 × 5 mL). The organic layers were pooled then concentrated *in vacuo* to afford the product as a dark brown oil (24.4 mg, 98%). LC–MS (*m*/*z*): 345.9 [M+Na]^+^; HPLC: *t*_R_ 7.075 min, >95% purity (214 and 254 nm); ^1^H NMR (400 MHz, CDCl_3_) *δ* 8.26 (d, *J* = 9.2 Hz, 1H), 7.69 (d, *J* = 8.4 Hz, 2H), 7.29 (d, *J* = 8.0 Hz, 2H), 7.18 (dd, *J* = 9.2 Hz, 2.4 Hz, 1H), 6.96 (d, *J* = 2.4 Hz, 1H), 4.80 (d, *J* = 2.4 Hz, 2H), 2.96 (sept, *J* = 6.8 Hz, 1H), 2.59 (t, *J* = 2.4 Hz, 1H), 1.27 (d, *J* = 7.2 Hz, 6H). ^13^C NMR (101 MHz CDCl_3_) *δ* 192.6 (C), 161.8 (C), 155.6 (C), 140.1 (C), 139.0 (C), 133.6 (C), 129.5 (CH), 114.6 (CH), 127.0 (CH), 115.9 (CH), 77.4 (CH), 77.3 (C), 56.6 (CH_2_), 34.4 (CH), 33.6 (CH_3_).

#### 4.2.4 (2-Amino-5-(prop-2-yn-1-yloxy)phenyl) (4-isopropylphenyl)methanone (**9**)^29^

Compound **8** (400 mg, 1.24 mmol, 1 eq.) was dissolved in ethanol (10 mL) followed by the addition of tin(II) chloride (1.70 g, 8.61 mmol, 7 eq.). The reaction was refluxed at 80 °C for 18–48 h. The reaction mixture was extracted with EtOAc (10 mL), washed two times with water, dried with anhydrous Na_2_SO_4_ and concentrated under reduced pressure. The resulting residue was purified by flash chromatography using (Petroleum spirits: EtOAc 6:1) to elute the product as a dark brown oil (267 mg, 74%). LC–MS (*m*/*z*): 294.1 [M+H]^+^; HPLC: *t*_R_ 6.430 min, >95% purity (214 and 254 nm); ^1^H NMR (400 MHz, CDCl_3_) *δ* 7.63 (d, *J* = 8.3 Hz, 2H), 7.30 (d, *J* = 8.2 Hz, 2H), 7.12 (d, *J* = 2.9 Hz, 1H), 7.04 (dd, *J* = 8.9, 2.9 Hz, 1H), 6.71 (d, *J* = 8.9 Hz, 1H), 4.53 (d, *J* = 2.4 Hz, 2H), 2.98 (hept, *J =* 6.9 Hz, 1H), 2.47 (t, *J* = 2.4 Hz, 1H), 1.29 (d, *J* = 6.9 Hz, 6H).

#### 4.2.5 (4-Isopropylphenyl) (2-((3-nitrobenzyl)amino)-5-(prop-2-yn-1-yloxy)phenyl)methanone (**10**)

Compound **9** (100 mg, 0.340 mmol, 1 eq.) was dissolved in 1,4-dioxane (5 mL), followed by the addition of 1-(bromomethyl)-3-nitrobenzene (147 mg, 0.680 mmol, 2 eq.) and *N*,*N*-diisopropylethylamine (119 μL, 0.680 mmol, 2 eq.). The reaction was heated to 80 °C and left to stir overnight. 1,4-Dioxane was removed *in vacuo* and the resulting residue was taken up in DCM (10 mL), washed with water (5 mL), dried over anhydrous Na_2_SO_4_ and then concentrated under reduced pressure. The crude reaction mixture was carried on to the next step without further purification. LC–MS (*m*/*z*): 429.2 [M+H]^+^.

#### 4.2.6 (4-Isopropylphenyl)-1-(3-nitrobenzyl)-6-(prop-2-yn-1-yloxy)quinazolin-2(1H)-one (**11**)

Compound **10** (146 mg, 0.340 mmol, 1 eq.) and sodium cyanate (33.2 mg, 0.511 mmol, 1.5 eq.) were dissolved in glacial AcOH (1 mL) and allowed to stir for 12 h. The reaction was neutralised with an aqueous solution of NaOH (1 mol/L), extracted with EtOAc (10 mL), dried with anhydrous Na_2_SO_4_ and concentrated under reduced pressure. The compound was purified *via* flash chromatography using EtOAc and petroleum spirits (1.5:1) to yield a yellow solid (55.6 mg, 36% over two steps). LC–MS (*m*/*z*): 454.2 [M+H]^+^; HR-ESMS Calcd. for C_27_H_23_N_3_O_4_^+^ [M+H]^+^ 454.1689, found 454.1702; HPLC *t*_R_ 6.204 min, >95% purity (214 and 254 nm). ^1^H NMR (400 MHz, CDCl_3_) *δ* 7.63 (d, *J* = 8.3 Hz, 2H), 7.30 (d, *J* = 8.2 Hz, 2H), 7.12 (d, *J* = 2.9 Hz, 1H), 7.04 (dd, *J* = 8.9, 2.9 Hz, 1H), 6.71 (d, *J* = 8.9 Hz, 1H), 4.53 (d, *J* = 2.4 Hz, 2H), 2.98 (hept, *J =* 6.9 Hz, 1H), 2.47 (t, *J* = 2.4 Hz, 1H), 1.29 (d, *J* = 6.9 Hz, 6H).

#### 4.2.7 (3-Aminobenzyl)-4-(4-isopropylphenyl)-6-(prop-2-yn-1-yloxy)quinazolin-2(1H)-one (**12**)

Synthesis as described in the procedure for **9** using compound **11** (65.9 mg, 0.144 mmol). The crude reaction mixture was carried on to the next step without further purification. LC–MS (*m*/*z*): 424.2 [M+H]^+^.

#### 4.2.8 4-(4-Isopropylphenyl)-1-(3-isothiocyanatobenzyl)-6-(prop-2-yn-1-yloxy)quinazolin-2(1H)-one (**4**)

A mixture of **12** (61.1 mg, 0.144 mmol), 1,1′-thiocarbonyldiimidazole (TCDI) (77.1 mg, 0.432 mmol) and DMAP (1.42 mg, 0.01 mmol) were dissolved in 3 mL dry THF under an N_2_ atmosphere. The reaction was stirred at room temperature for 2 h after which the THF was removed under reduced pressure. The resulting residue was purified *via* flash chromatography using petroleum spirits and EtOAc (2:1) to furnish a yellow solid (22.6 mg, 34% over two steps). LC–MS (*m*/*z*): 466.1 [M+H]^+^; HR-ESMS Calcd. for C_28_H_24_N_3_O_2_S^+^ [M+H]^+^ 466.1511, found 466.1521; HPLC *t*_R_ 6.768 min, >95% purity (214 and 254 nm). ^1^H NMR (400 MHz, CDCl_3_) *δ*. 7.76 (d, *J* = 8.2 Hz, 2H), 7.53 (d, *J* = 2.9 Hz, 1H), 7.39 (d, *J* = 8.2 Hz, 2H), 7.34 (dd, *J* = 9.2, 2.9 Hz, 1H), 7.29 – 7.34 (m, 1H), 7.22 – 7.26 (m, 1H), 7.18 (d, *J* = 9.3 Hz, 1H), 7.11–7.17 (m, 2H), 5.51 (s, 3H), 4.65 (d, *J* = 2.4 Hz, 3H), 3.02 (hept, *J* = 6.9 Hz, 1H), 2.55 (t, *J* = 2.4 Hz, 1H), 1.32 (d, *J* = 6.9 Hz, 6H). ^13^C NMR (101 MHz, CDCl_3_) *δ*. 174.7 (C), 155.9 (C), 152.5 (C), 152.4 (C), 138.7 (C), 138.1 (C), 135.6 (C), 133.9 (C), 132.1 (C), 130.4 (CH), 130.1 (CH), 126.8 (CH), 126.0 (CH), 125.3 (CH), 125.0 (CH), 124.1 (CH), 116.9 (CH), 116.1 (CH), 113.8 (CH), 78.0 (CH), 76.5 (C), 56.5 (CH_2_), 47.4 (CH_2_), 34.3 (CH), 24.0 (CH_3_).

### 4.3 Pharmacology

#### 4.3.1 Materials

Flp-In™ TREX™ Human Embryonic Kidney (HEK) 293 cells were purchased from Invitrogen (Carlsbad, USA). Fluo-8-AM (acetoxymethyl ester) was purchased from Abcam. Ionomycin was purchased from Cayman Chemicals (Michigan, USA). ATF936, Dulbecco's Modified Eagle's Medium (DMEM), poly-d-lysine, hygromycin B, blasticidin HCl, tetracycline and all other reagents were purchased from Sigma–Aldrich (St. Louis, USA). Phosphate buffered saline solution (PBS) was prepared by dissolving 137 mmol/L NaCl, 2.7 mmol/L KCl, 10 mmol/L Na_2_HPO_4_, 1.8 mmol/L KH_2_PO_4_ in 1 L at pH 7.4.

#### 4.3.2 Cell lines

FlpIn TRex HEK293 cells stably expressing the cmyc-tagged WT CaSR have been described previously^12^^,^^33^ and were maintained in DMEM supplemented with 5% FBS and antibiotic selection (200 μg/mL hygromycin; 5 μg/mL blasticidin) at 37 °C in a humidified atmosphere of 5% CO_2_ and 95% O_2_.

#### 4.3.3 Cell culture

Flp-In HEK293-TREx c-myc-CaSR cells were maintained in DMEM supplemented with 200 μg/mL hygromycin B, 5% tetracycline-free FBS, and 5 μg/mL blasticidin at 37 °C in a humidified atmosphere of 5% CO_2_ and 95% O_2_. Flp-In HEK293 non-transfected cells were maintained in DMEM supplemented with 5% tetracycline-free FBS at 37 °C in a humidified atmosphere of 5% CO_2_ and 95% O_2_. Cells were seeded into clear 96-well plates, which were coated with 2.5 μg per well poly-d-lysine to facilitate cell adherence. Cells were harvested with 2 mmol/L EDTA in PBS, centrifuged (3 min, 350 g), resuspended in DMEM and seeded at a density of 20,000 cells per well for non-transfected cells and at 40,000 cells per well for c-myc-CaSR cells for Ca^2+^_i_ mobilization assays. 300 ng/mL tetracycline was added to the c-myc-CaSR cells 18–21 h before the assays were conducted to obtain maximum CaSR expression.

#### 4.3.4 Ca^2+^_i_ mobilization assay

Cells were washed with assay buffer (150 mmol/L NaCl, 2.6 mmol/L KCl, 1.18 mmol/L MgCl_2_, 10 mmol/L d-glucose, 10 mmol/L HEPES, 0.1 mmol/L Ca^2+^, 0.5% BSA and 4 mmol/L probenecid at pH 7.4) and loaded with Fluo-8 AM (1 μmol/L) for 1 h at 37 °C. For functional interaction studies between NAMs and Ca^2+^_o_ each cell plate was washed with 80 μL of assay buffer before the addition of 10 μL of modulator of interest 30 min prior to the addition of Ca^2+^_o_. Each well was treated with 10 μL of a single agonist and/or modulator concentration. For the washout assay, cells were washed with 100 μL of washout assay buffer (150 mmol/L NaCl, 2.6 mmol/L KCl, 10 mmol/L d-glucose, 10 mmol/L HEPES, 0.5 mmol/L EGTA, 0.1% BSA, NaOH at pH 7.4) before the addition of 90 μL of assay buffer and 10 μL of a single modulator concentration and incubated for 2 h at 37 °C. Cells were washed with 3 × 50 μL of wash buffer (150 mmol/L NaCl, 2.6 mmol/L KCl, 10 mmol/L d-glucose, 10 mmol/L HEPES, 0.5 mmol/L EGTA, 0.1% BSA, NaOH at pH 7.4) removing the wash buffer between each addition. Cells were washed with 200 μL of wash buffer and incubated at 37 °C for 30 min. This process was repeated an additional 3 times before cells were washed with 100 μL assay buffer and loaded with 50 μL Fluo-8 AM (1 μmol/L) for 1 h at 37 °C. The release of Ca^2+^_i_ was measured at 37 °C using a FDSS/μCELL Functional Drug Screening System (Hamamatsu Photonics; Hamamatsu City, Shizouka Prefecture, Japan). Fluorescence was detected for 15 s to establish a baseline response and then a further 60 s at 485 nm excitation and 525 nm emission, and the peak Ca^2+^_i_ mobilization response was used to determine the agonist response. Relative peak fluorescence units were normalized to the fluorescence stimulated by the maximal response of Ca^2+^_o._

#### 4.3.5 *In vivo* studies in rats

All studies were conducted in 6–8 week old male Sprague Dawley rats, according to experimental protocols approved by the Monash Institute of Pharmaceutical Sciences Animal Ethics Committee (Protocol 26791). Briefly, vascular cannulations (left carotid artery (for blood sampling) and jugular vein (for IV administration)) were performed on the day prior to dosing and animals after which animals were allowed to recover overnight with *ad lib* access to water in Raturn™ metabolic cages connected to a Culex ABS™ automated blood sampler (BASi, West Lafayette, USA). Each rat was administered a single compound at a dose of 0.61 mg/kg as a 10 min constant-rate intravenous infusion. Samples of blood were taken at the middle and end of the infusion (5 and 10 min) and then intermittently to 370 min (15, 20, 25, 30, 40, 70, 160, 240 and 370 min).

Plasma was separated from blood and concentrations of ATF936 or **4** were determined *via* LC–MS/MS using assays validated for specificity, accuracy and precision. PTH concentrations in the plasma were determined using the Immunotopics rat intact PTH ELISA. Plasma from rats administered with ATF isothiocyanate (**4**) was diluted 1:2 (15 μL plasma diluted in 15 μL ddH_2_O). 25 μL diluted plasma, PTH standard or PTH control were added into wells of the ELISA plate. 100 μL antibody solution (1:1 mix of 1-part PTH biotinylated antibody mixed with 1-part PTH HRP conjugated antibody) was added to each well. The ELISA plate was covered and sealed with a top seal followed by foil and incubated for 3 h at room temperature on a rocking platform. The solution was aspirated (flicked) from wells and each well was washed five times with 350 μL wash solution. 150 μL HRP substrate was added to each well and the plate was sealed with top seal and foil and incubated for 30 min at room temperature on a rocker. Absorbance was measured at 620 nm on both the Flexstation 3 and the Envision. 100 μL stop solution was added to each well and the plate was incubated for 1 min prior to measuring absorbance at 450 nm (within 10 min of adding stop solution) on the Flexstation 3 and the Envision.

#### 4.3.6 Data and statistical analysis

Data describing the interaction between Ca^2+^_o_ and the NAMs were fitted to the following variants of the operational model of cooperative agonism and allosterism, as shown in Eq. (1) ^32^:

(1) $$Effect=\frac{E_{m}{\left[\tau_{A}{\left[A+C\right]}^{n_{B}}\left(K_{B}+\alpha \beta \left[B\right]\right)+\tau_{B}\left[B\right]{\left[K_{B}\right]}^{n_{B}}\right]}^{n_{T}}}{{\left({\left[A+C\right]}^{n_{B}}K_{B}{+K}_{A}^{n_{B}}K_{B}{+K}_{A}^{n_{B}}\left[B\right]+\alpha {\left[A+C\right]}^{n_{B}}\left[B\right]\right)}^{n_{T}}+{\left[{\left[A\right]}^{n_{B}}\left(K_{B}+\alpha \beta \left[B\right]\right)+\tau_{B}\left[B\right]K_{A}^{n_{B}}\right]}^{n_{T}}}$$

where *K*_B_ is the affinity of the allosteric ligand; $\tau_{A}$ and $\tau_{B}$ are the operational efficacies of the orthosteric agonist and allosteric ligand, respectively; *α* and *β* are the allosteric effects on orthosteric agonist affinity and efficacy, respectively; [A] and [B] are the molar orthosteric agonist and allosteric ligand concentrations, respectively; *E*_m_ is the maximal system response; $n_{T}$ is the transducer slope linking agonist concentration to response; $n_{B}$ is the binding slope linking agonist concentration to occupancy; and [C] is the contaminating Ca^2+^_o_ concentration present in the assay buffer set equal to 0.1 mmol/L. In all instances, $n_{T}$ was constrained to unity, *α* was constrained to zero, $\tau_{B}$ was constrained to −1000, $K_{B}$ was constrained to be less than zero but greater than 10 nmol/L and all other parameters were shared between all data sets.

Or the comparative version, which is a simplified variant, as showen in Eq. (2) ^32^:

(2) $$Effect=\frac{E_{m}{\left[\tau_{A}\left[A+C\right]\left(K_{B}+\alpha \beta \left[B\right]\right)+\tau_{B}\left[B\right]{\left[{EC}_{50}\right]}^{n_{B}}\right]}^{n_{T}}}{{{\left[{EC}_{50}\right]}^{n_{B}nT}{\left(K_{B}+\left[B\right]\right)}^{nT}+\left({\left[A+C\right]}^{n_{B}}\left(K_{B}+\alpha \beta \left[B\right]\right)+\tau_{B}\left[B\right]{\left[{EC}_{50}\right]}^{n_{B}}\right)}^{n_{T}}}$$

where *K*_B,_ *α*, *β, E*_*m*_*, n*_*T*_*, n*_*B*_*,* [A], [B] and [C] are defined as above for equation 1, with the same constraints applied. LogEC_50_ is the concentration of agonist (Ca^2+^_o_) that gives a half-maximal response. Non-linear regression analysis was performed in GraphPad Prism 10.1.2.

Data describing between Ca^2+^_o_ and the NAMs were additionally fitted to a four-parameter dose-response curve, as showen in Eq. (3):

(3) $$\text{Effect}=\frac{\text{Bottom}+\left(\text{Top}-\text{Bottom}\right)}{(1+10^{\left({\text{LogEC}}_{50}-X\right)*\text{HillSlope})})}$$

where Top and Bottom are the plateaus in the *y*-axis, LogEC_50_ is the concentration of agonist that gives a response that is half way between Bottom and Top and HillSlope describes the steepness of the curves. A one-way ANOVA with Dunnett's multiple comparisons post-test was used to determine statistical differences between logEC_50_, *E*_max_ and Hill slope. A repeated measures two-way ANOVA with multiple comparisons was used to determine statistical differences between intact PTH concentrations. An unpaired two-tailed *t*-test was used to determine statistical differences between the AUC values.

### 4.4 Computational modelling

Structural information obtained from the Cryo-EM PDBID:7M3E^28^ was used as the basis for docking. Incomplete regions in the pdb were rebuilt and modelled using Monte Carlo optimization in internal coordinates using ICM. Docking was performed using ICM, where the candidate was placed in the 7TM binding pocket that contained the NAM NPS2143 (**1**). NAMs were initially placed in the center of the presumed binding site before extensive biased probability Monte Carlo sampling in internal coordinates. Therefore, the hydrogen bonding, van der Waals, and hydrophobic and electrostatic potential of the CaSR 7TM binding cavity were calculated to create a “grid potential map,” which was used to score the binding potential of randomized conformations of the NAM. Binding scores were calculated as described previously^34^. Ten poses with the lowest calculated binding scores were retained for each NAM. These poses were further refined using a “flexible receptor” approach, by undertaking biased probability Monte Carlo optimization of receptor residue sidechains^35^, while simultaneously sampling NAM shape and position by Monte Carlo randomization. Final results were assessed using a combination of ICM docking scores and calculated binding energies^36^^,^^37^ coupled with manual inspection for agreement with mutagenesis data and with published structure activity relationship (SAR) data.

## Author contributions

Jesse Dangerfield: Writing – original draft, Methodology, Investigation, Formal analysis, Data curation, Conceptualization. Aaron DeBono: Writing – review & editing, Supervision, Project administration, Methodology. Andrew N. Keller: Writing – review & editing, Supervision, Methodology. Tracy M. Josephs: Writing – review & editing, Methodology. David M. Shackleford: Writing – review & editing, Methodology, Data curation. Karen J. Gregory: Writing – review & editing, Supervision, Software, Project administration, Funding acquisition, Data curation, Conceptualization. Katie Leach: Writing – review & editing, Supervision, Project administration, Methodology, Funding acquisition, Data curation, Conceptualization. Ben Capuano: Writing – review & editing, Supervision, Project administration, Methodology, Conceptualization.

## Conflicts of interest

The authors declare no conflicts of interest.

## Appendix A Supporting information

The following is the Supporting information to this article:
