# Supplementary material for: A novel chemoreactive calcilytic for the potential treatment of autosomal dominant hypocalcemia
Source: Acta Pharm Sin B. 2025 Aug 7;15(10):5387–99. doi: 10.1016/j.apsb.2025.07.044 (PMC12541615; doi:10.1016/j.apsb.2025.07.044)
Supplement: Multimedia component 1 [file mmc1.pdf]

Supporting Information for

Original article

A novel chemoreactive calcilytic for the potential treatment of autosomal dominant hypocalcemia

Jesse Dangerfield[a,b], Aaron DeBono[a], Andrew N Keller[b], Tracy M Josephs[b,c], David M Shackleford[d], Karen J Gregory[b,c,*], Katie Leach[b,c,*], Ben Capuano[a,*]

[a]*Medicinal Chemistry, Monash Institute of Pharmaceutical Sciences, Monash University, VIC 3052, Australia*

[b]*Drug Discovery Biology, Monash Institute of Pharmaceutical Sciences, Monash University, VIC 3052, Australia*

[c]*Australian Research Council Centre for Cryo-electron Microscopy of Membrane Proteins, Monash University, VIC 3052, Australia*

[d]*Centre for Drug Candidate Optimisation, Monash Institute of Pharmaceutical Sciences, Monash University, VIC 3052, Australia*

Received 2 December 2024; received in revised form 10 May 2025; accepted 12 June 2025

[*]Corresponding authors.

E-mail addresses: karen.gregory@monash.edu (Karen J Gregory), katie.leach@monash.edu (Katie Leach), ben.capuano@monash.edu (Ben Capuano)

[†]These authors made equal contributions to this work.

Computational modelling

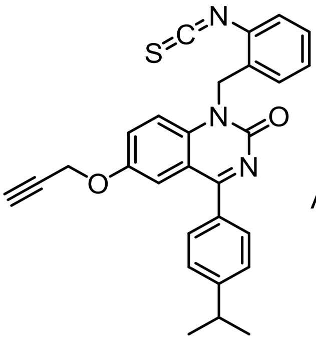

ATF936-2'-NCS

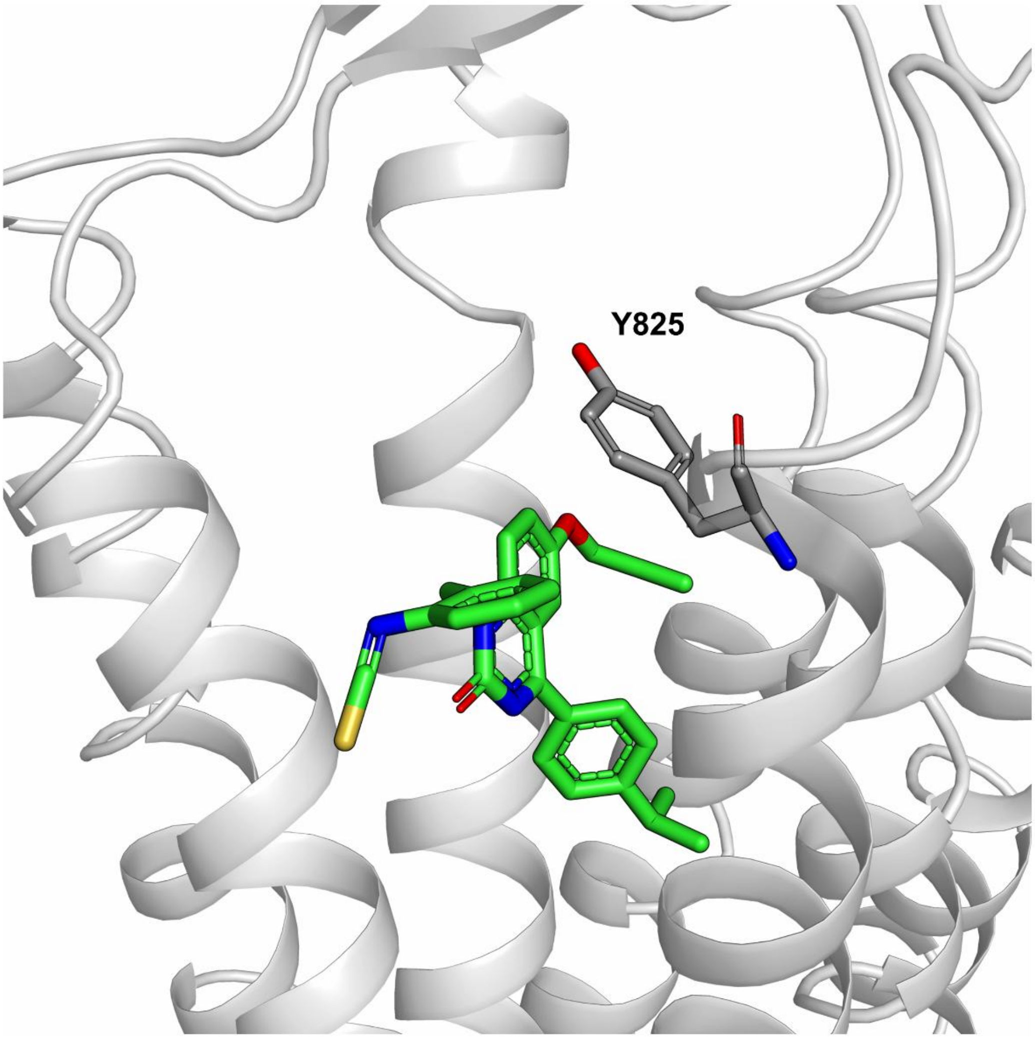

Figure S1 The chemical structure of ATF936-2′-NCS (top) and the binding pose of ATF936-2′-NCS in the CaSR cryo-EM structure 7M3E (bottom).

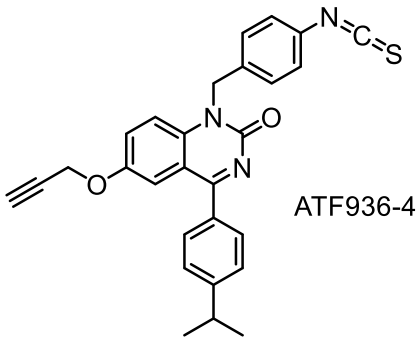

ATF936-4'-NCS

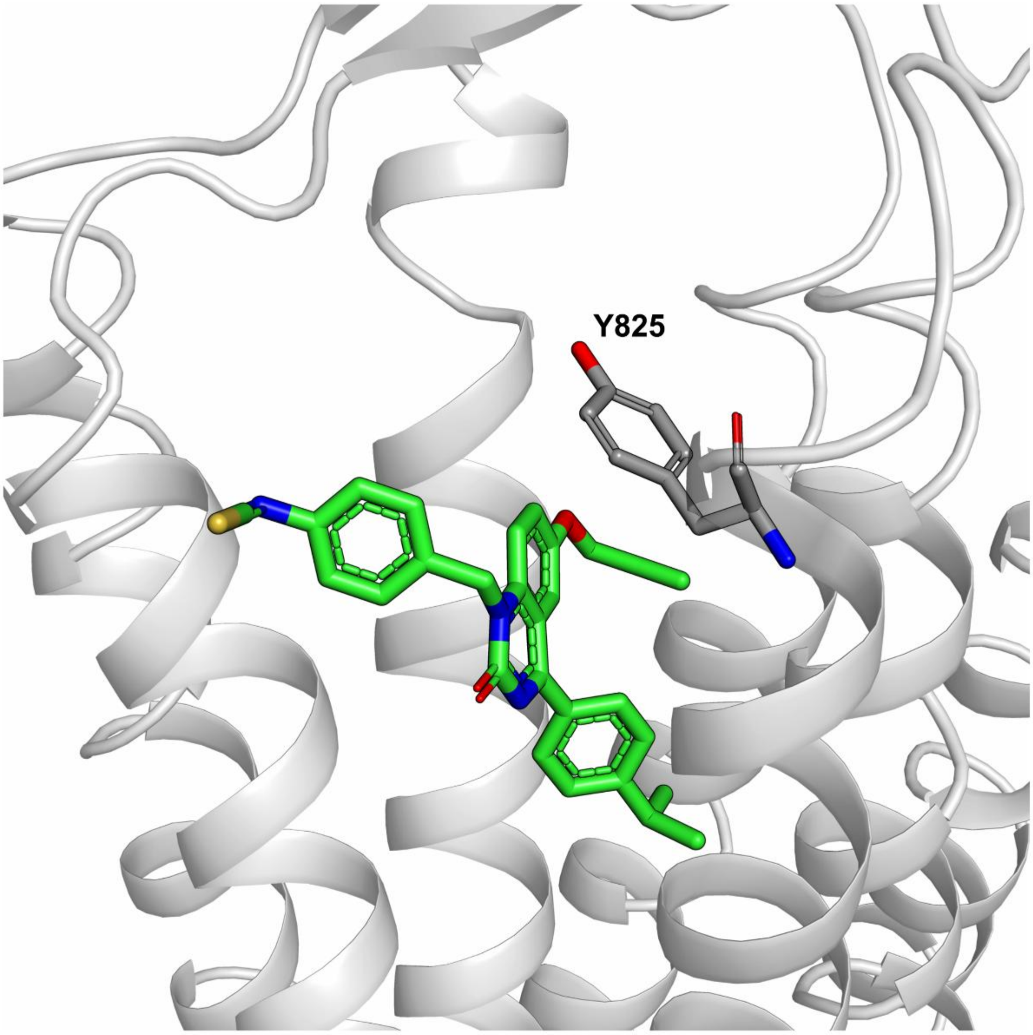

Figure S2 The chemical structure of ATF936-4′-NCS (top) and the binding pose of ATF936-4′-NCS in the CaSR cryo-EM structure 7M3E (bottom).

Chemistry

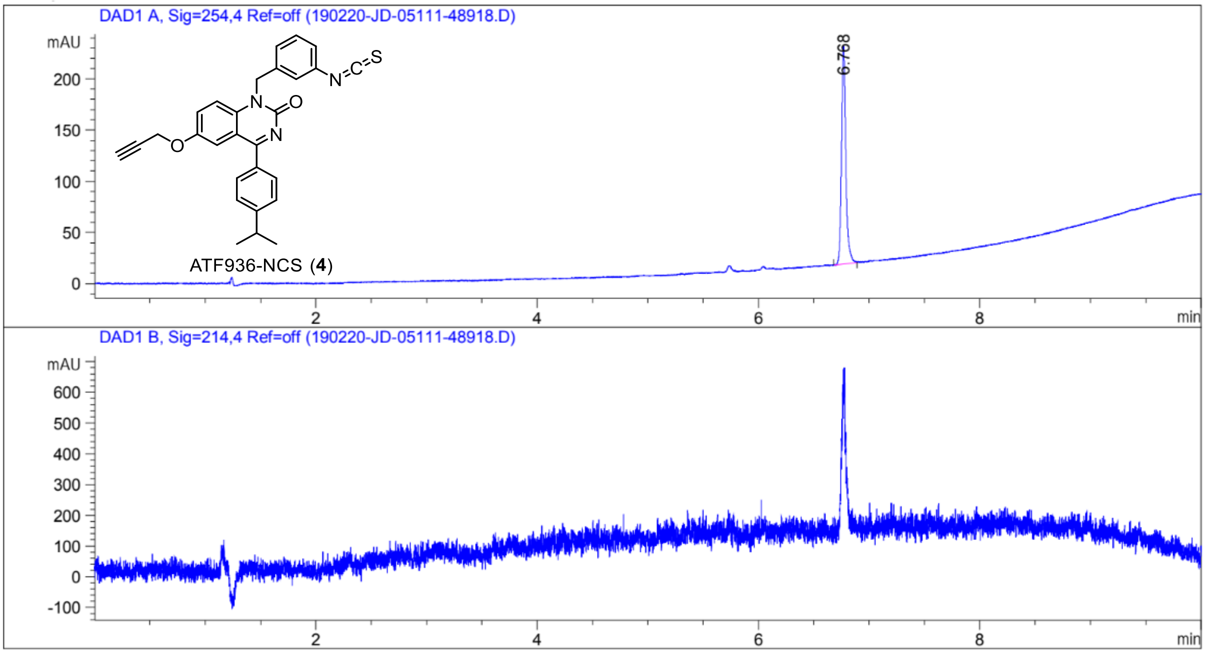

Figure S3.1 Analytical HPLC chromatograms for ATF936-NCS (**4**) using UV detection at 254 nm (upper) and 214 nm (lower).

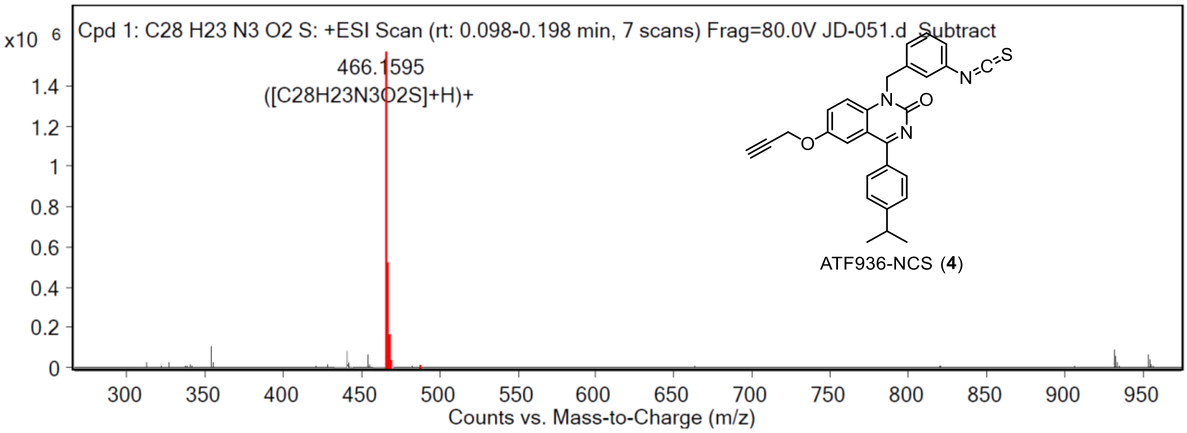

Figure S3.2 High-resolution mass spectrum for ATF936-NCS (**4**).

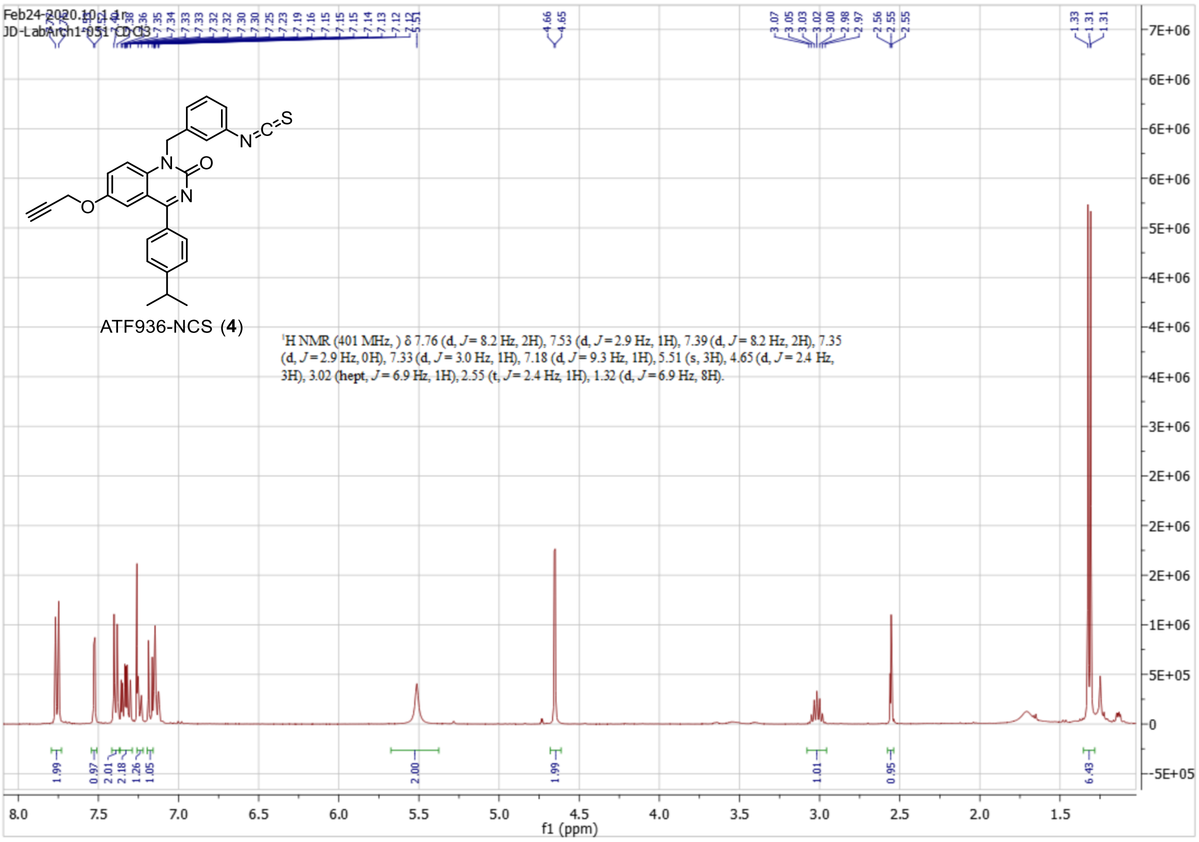

Figure S3.3 ^{1}H NMR spectrum for ATF936-NCS (**4**) in $CDCl_3$.

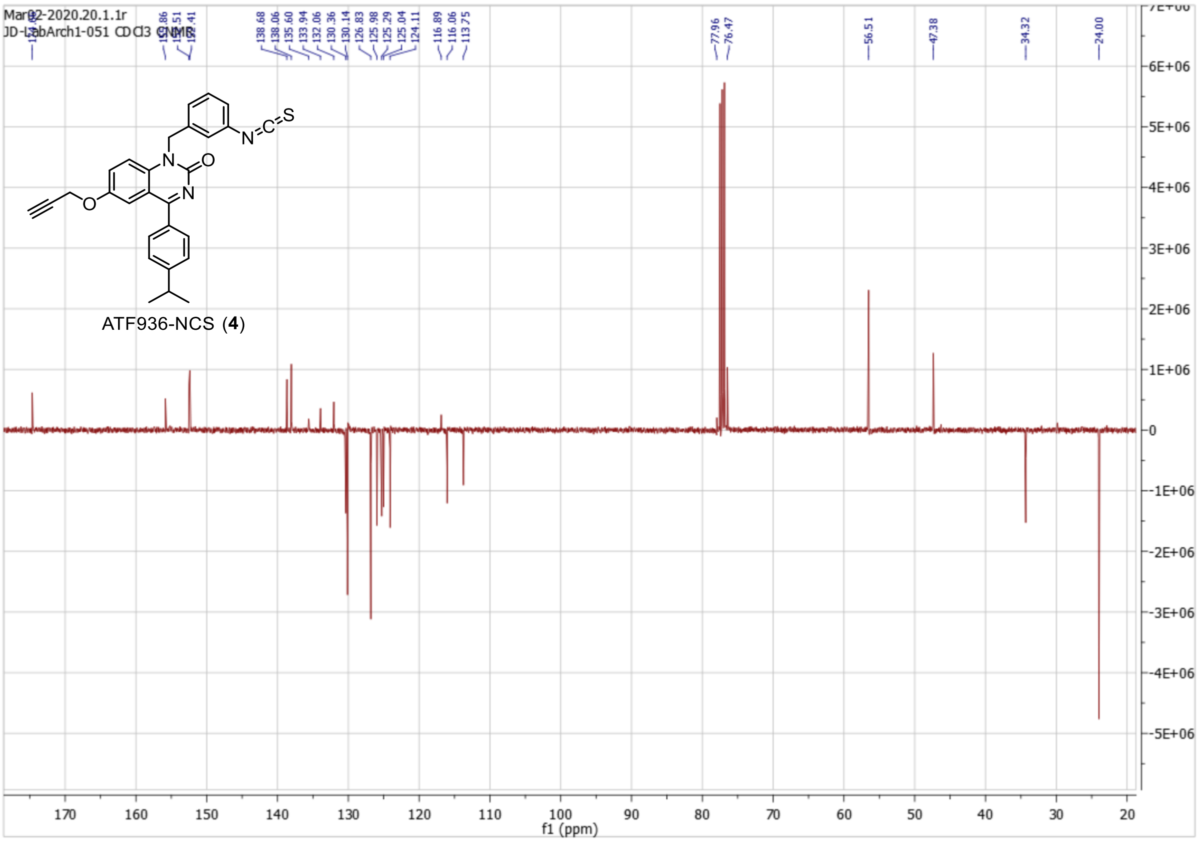

Figure S3.4 ^{13}C NMR spectrum for ATF936-NCS (**4**) in $CDCl_3$.
